# Antibiotics Prophylaxis Practice in Arthroplasty Surgeries

**DOI:** 10.7759/cureus.54075

**Published:** 2024-02-12

**Authors:** Emmanuel O Oladeji, Adedoyin M Wusu, Ahmed Lashin, Ahmed Kaddah, Oghofori Obakponovwe, Madhu Rao

**Affiliations:** 1 Department of Trauma and Orthopedics, St. Richard's Hospital, Chichester, GBR

**Keywords:** joint replacement, audit, pji, prosthetic joint infection, surgical site infection, antibiotic prophylaxis

## Abstract

Background

Infection in orthopedic surgery is one of the most dreaded complications. It is associated with prolonged morbidity, disability, and increased mortality. One of the cornerstones of the prevention of infections is antibiotic prophylaxis. This study assessed the practice of antibiotic prophylaxis in arthroplasty surgeries in our local hospital.

Methods

One hundred and seventy-one elective joint replacement patients were retrospectively analyzed for documentation of antibiotic plan in postoperative instruction, choice of antibiotic, dose, and dosage. Compliance with the dosage (duration and frequency) of antibiotic prophylaxis was compared among patients who underwent different operations, among patients whose operation notes had antibiotics plans, and among those patients whose operation notes lacked this information.

Results

Ninety-six females and 75 males with a mean age of 71.4±9.8 years who underwent hip replacement, knee replacement, or shoulder replacement were included in this study. Preoperative and postoperative antibiotics were received by 100% and 94.7% of patients, respectively. In 19.3%, there was no instruction about postoperative antibiotics while 4% missed at least one postoperative dose. The dosage of postoperative prophylactic antibiotics was variable as 26.3% of the patients experienced delayed administration of doses. Not having intravenous access, failure to prescribe antibiotics, and prescribing antibiotics in the "once only" rather than "regular medication" section of the medication chart were the reasons for improper timing of antibiotic doses. Observing surgical safety checklist was effective in ensuring preoperative antibiotic administration, whereas failing to document antibiotic plan in operation note was associated with poor compliance with postoperative dosage. Interprofessional participation is crucial to compliance with antibiotic prophylaxis practice.

Conclusion

This study identified key areas for improvement in our antibiotics prophylaxis practice. It resulted in implementing strategies to improve staff's awareness about the importance of timely administration of prophylactic antibiotics and proper documentation by all team members.

## Introduction

Prosthetic joint infection (PJI) is one of the most dreaded complications following a joint replacement. It is devastating for both the patient and the surgeon, often requiring further operations, a long course of antibiotics, and longer hospital stays, leading to increased morbidity and mortality and huge costs associated with treatment [1]. About 15% of revisions for hip or knee arthroplasty are due to infections and the associated five-year mortality could be as high as 25.9% [2-4].

The use of prophylactic antibiotics is one of the cornerstones of preventing infection following joint replacement surgery [5]. Their effectiveness in reducing the risk of prosthetic joint infection was first established in a landmark study by Lidwell et al. and has been corroborated by recent studies [6,7].

Practice regarding the choice of antimicrobial agent and duration of prophylactic regime varies with local epidemiology and surveillance, patient factors, and surgeon’s preference. Regardless of the choice, the literature is unanimous about the preference for bactericidal antimicrobial agents and the 24-hour threshold for administration, beyond which continuation of prophylactic antibiotics has not been proven to reduce the rate of infection [7].

Regardless of the choice of antibiotics, achieving and sustaining the minimum inhibitory concentration (MIC) of antibiotics is crucial to maintaining antimicrobial susceptibility of microbes, this is accomplished by administering the correct dose at the right dosage. The understanding that the initial two hours following an incision are critical for the potential introduction of pathogens emphasizes the importance of promptly administering prophylactic antibiotics. Typically, these antibiotics should be given within 60 minutes before the surgical incision [8-10].

Surveillance of adherence to these guidelines is essential to ensure standards are maintained and patient outcomes are not jeopardized. This study was conducted to ascertain the timing and duration of prophylactic antibiotics in joint replacement surgeries, against our local guidance.

## Materials and methods

This audit was a cross-sectional retrospective analysis of antibiotics prophylaxis practice in elective arthroplasty surgeries at a District General Hospital with a large volume of elective orthopedic surgeries. The study population is comprised of all consecutive patients who underwent elective arthroplasty surgeries between March 1, 2022, and June 30, 2022. A list of applicable patients was obtained from the elective surgery register of the trauma and orthopedics department and relevant data were sourced from the operation and anesthetic notes and the drug charts available on the secure online medical record platform.

We collected data on patients’ demographic characteristics, type of surgery, antibiotic received, timing of administration of antibiotics, the doses administered, the intervals between these doses, number of doses received, and documentation of antibiotic plan in the postoperative instruction. Patients undergoing revision arthroplasty surgeries for infection or receiving antibiotics for other indications preoperatively were excluded. Audit approval was obtained from the local clinical audit department and the project was carried out as a retrospective audit for the purpose of quality improvement, hence ethical approval was not required.

The audit was conducted against our local guidelines on antibiotic prophylaxis in orthopedic surgery and international best practices [10]. The prophylactic antibiotic regimen in our hospital is weight-based intravenous flucloxacillin and gentamicin administered within one hour prior to incision or knife to skin (KTS), and three subsequent doses of intravenous flucloxacillin at six hours, 12 hours, and 18 hours postoperatively. Flucloxacillin is replaced with teicoplanin in patients with penicillin allergy with no postoperative doses required.

Data extraction and analysis were conducted using Microsoft Excel software (Redmond, WA: Microsoft Corporation) and JASP software version 0.18 (Amsterdam, Netherlands: University of Amsterdam), respectively. ANOVA analysis was used to compare compliance with the timing of antibiotic prophylaxis among patients who underwent hip, knee, or shoulder arthroplasty. Chi-square analysis was performed to compare compliance with the timing of antibiotic doses among patients whose operation notes had antibiotics plans and those who did not. Significance was considered at the p<0.05 level. The findings of the audit were presented at the departmental clinical governance meeting where learning points and strategies to improve practice were discussed.

## Results

A total of 171 patients who underwent elective joint replacement were included in this study. Ninety-three had a hip replacement, 71 knee replacement, and seven received a shoulder replacement. A total of 54.7% were females and the mean age was 71.4±9.8 years. A summary of prophylactic antibiotic practices is shown in Table 1. Intravenous flucloxacillin was the most commonly used antibiotic and all the patients received the appropriate dosage. Preoperative and postoperative antibiotics were received by 100% and 94.7% patients, respectively. In 22.8%, there was no instruction about the antibiotics plan in the operation note while 4% missed at least one dose, and the third postoperative dose was the most commonly missed.

**Table 1:** Antibiotics prophylaxis practice. preop: preoperative; postop: postoperative; op note: operation note

Variables	Frequency (%)
Received preop antibiotics	
Yes	171 (100)
No	0 (0)
Received complete course of postop antibiotics (N=154)	
Yes	146 (94.7)
No	9 (5.3)
Antibiotics received	
Flucloxacillin	154 (90.1)
Gentamicin	171 (100)
Teicoplanin	17 (9.9)
Antibiotics plan documented in op note	
Yes	132 (77.2)
No	39 (22.8)
Missed postop doses (N=9)	
First dose	0
Second dose	2 (22.2)
Third dose	7 (77.8)
All the postop doses	0
Delay in postop dose administration	
No	98 (63.6)
Yes	56 (36.4)
Postop dose delayed (N=56)	
First dose	29 (51.8)
Second dose	11 (19.6)
Third dose	16 (28.6)

The mean time between preoperative antibiotic prophylaxis and KTS for all the patients was 26.7±12 minutes (Table 2). The mean time was 24.5±13.4 minutes for patients who underwent knee replacement, 26.3±12.9 minutes for those who had hip replacement, and 28.3±2.4 for patients who had shoulder replacement; however, ANOVA analysis showed no statistical significance among the subgroups.

**Table 2:** Timing of prophylactic antibiotic administration. preop: preoperative; postop: postoperative

Variables	Frequency (%)
Preop antibiotics (minutes) (N=137)	
0-14	27 (19.7)
15-29	53 (38.7)
30-44	37 (27.0)
45-60	18 (13.1)
>60	2 (1.5)
Mean±SD	26.7±12
Delay in postop dose administration (hours) (N=56)	
<1	1 (1.8)
1-2	9 (16.1)
2-3	41 (73.2)
3-4	2 (3.6)
>4	3 (5.3)

**Figure 1:**
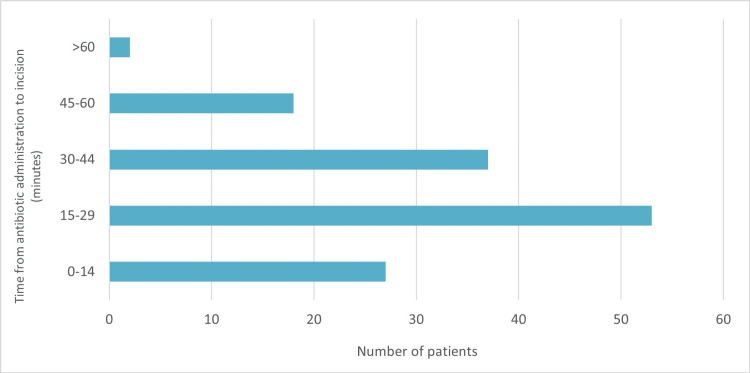
Time from antibiotic administration to incision.

While a vast majority of the patients received preoperative prophylactic antibiotics within 45 minutes before incision (Figure 1), the dosage of postoperative antibiotics was variable, as 29.2% experienced a 1-12 hours delay in administration of doses (Table 2).

**Figure 2:**
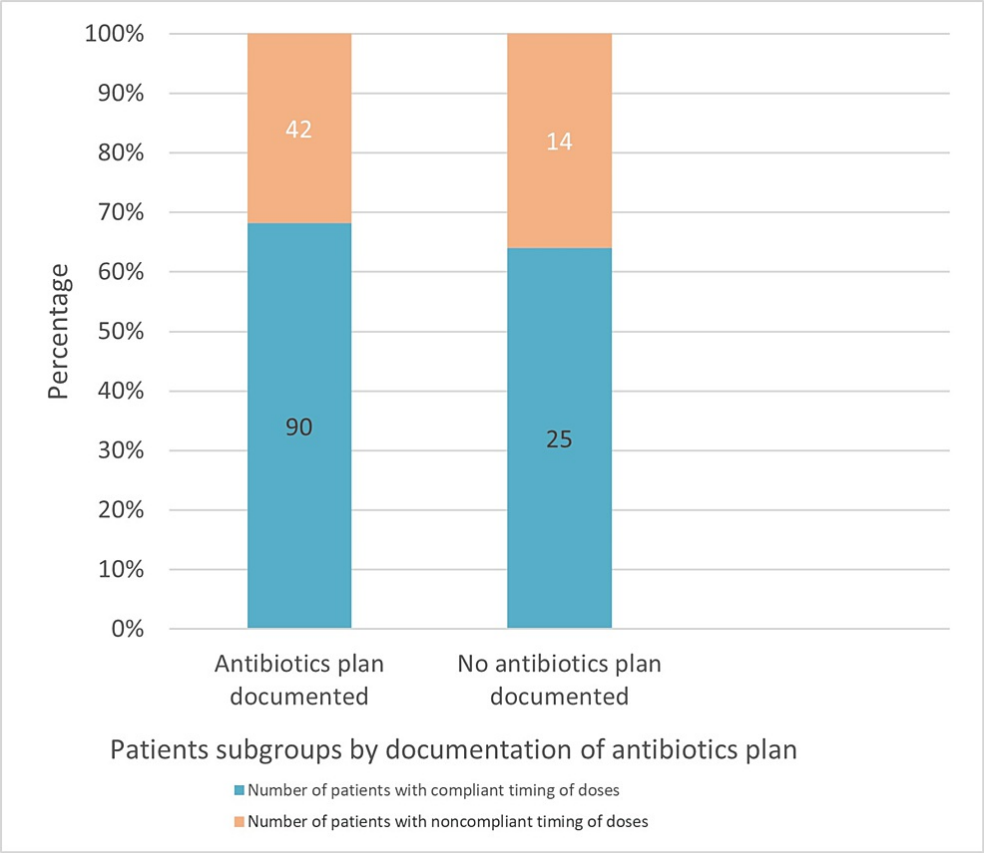
Timing of postoperative dose based on documentation of antibiotics plan.

Out of the 56 patients who experienced a delay in antibiotic administration, 31 underwent hip replacement and 23 had knee replacement, but no statistical significance was demonstrated. Those who had no antibiotic plan documented in their operation note experienced a higher rate of noncompliance with the timing of postoperative doses (35.9% versus 31.8%) but this difference did not reach statistical significance (Figure 2).

The most frequently delayed dose was the first postoperative dose while the third postoperative doses were the most missed (Table 1). The reasons for improper timing of antibiotic doses included the following: intravenous access not available, antibiotics not prescribed, or antibiotic doses being prescribed in the "once only" rather than "regular medication" section of the medication chart. Seven patients received more doses of antibiotics than required; four had intravenous flucloxacillin administered up to 36 hours postoperatively, while the three other patients received postoperative doses of teicoplanin contrary to the local guidance.

## Discussion

The rational, safe, and effective use of antimicrobial agents remains one of the crucial strategies to prevent the devastating consequences of prosthetic joint infections. Surveillance of antibiotic prophylaxis practice is vital to ensure that standards are maintained, and to identify any variations in practice that may put patients at increased risk of PJI.

From our study, the utilization of flucloxacillin for antibiotic prophylaxis in most patients mirrors the national preference for penicillinase-resistant penicillin in trauma and orthopedics patients in the United Kingdom [11]. Flucloxacillin offers good cover against *Staphylococcus aureus*, the most implicated pathogen in prosthetic joint infection, and is commonly used for prophylactic regimens, often in combination with gentamicin [12]. Nevertheless, other studies have reported coagulase-negative staphylococci (CoNS) as the most prevalent microorganism in PJIs [13,14].

Administration of prophylactic antibiotics within a 60-minute window before incision is a proven strategy for reducing the risk of surgical site infection; this practice is encouraged by our hospital guidance [9]. Preoperative antibiotic administration was compliant with this guideline in 98.5% of patients. Our local protocol of observing the WHO Surgical Safety Checklist has proven to be an effective way of ensuring the timely administration of preoperative antibiotics. Compliance with this practice ensures that the minimum inhibitory concentration (MIC) of the antibiotic is achieved before KTS, allowing the antimicrobial agent sufficient time for tissue penetration to have its bactericidal effect on any contaminating microbe.

The pharmacodynamic impact of the appropriate timing of the preoperative prophylactic dose is just as crucial as the interval between it and subsequent doses. It has been demonstrated to have an effect on clinical efficacy and the propensity of bacteria to develop antibiotic resistance [15]. About one-quarter of the patients in this study experienced a delay in the administration of postoperative doses, the first postoperative dose being the most affected and the most frequently reported reason being lack of intravenous access or failing to prescribe antibiotics. The reasons reported for improper timing were similar to findings by Thonse et al., they additionally reported failure to indicate timing of doses and lack of trained staff to administer antibiotics among a cohort of patients who underwent hip fracture surgery [16].

Overall, the duration of administration of postoperative doses of prophylactic antibiotics in most patients was restricted to the 24-hour limit stipulated by our local guidance and recommended by literature [7,17]. However, it is pertinent to note that antibiotic prophylaxis for extended duration has been advocated by some authors in a subset of patients with high-risk characteristics which include diabetes, high body mass index, and active smokers, suggesting that the duration of antibiotic prophylaxis should be individualized and evidence-based and not just arbitrary and stereotypic [18]. Additionally, the operating surgeon must document postoperative instructions, as entrenched in Good Surgical Practice [19]. Regardless of the chosen duration, antibiotic prescription needs to be done safely to minimize their potential toxic effect, reduce the risk of developing resistant microorganisms, and avoid the morbidity associated with antibiotic misuse [20].

Through this study, there has been increased awareness among staff about the importance of prophylactic use of antibiotics and the multidisciplinary participation of the surgical, anesthetic, nursing, and infection control teams, towards ensuring that standards are maintained. Strategies to ensure timely administration, and promote proper and accurate documentation by all members of the team were highlighted. This includes introducing a ward transfer checklist that ensures postoperative antibiotics have been prescribed and patient has functioning intravenous access, ensuring that antibiotics are administered according to the regimen, and the appropriate escalation mechanisms to explore whenever there is noncompliance. The use of ward transfer checklist is particularly key, given the disproportionately high rate of delay reported with respect to the timing of first postoperative doses.

The limitations of this study are that this is a retrospective audit study with limited data obtained by its nature, and potentially subject to missing information and inaccurate details. The findings described may not be generalizable to other populations and need a full-scale study to properly account for possible confounders and undertake an objective test of association. Nevertheless, the study offers valuable insights on key aspects of antibiotic prophylaxis which lie at the core of modern surgical practice.

## Conclusions

This study has identified key aspects of our antibiotic prophylactic practice that require improvement and has generated targeted interprofessional action plans to improve patient care. Results of this study have demonstrated the effectiveness of surgical time-out in ensuring appropriate timing of preoperative antibiotic dose, the importance of documenting postoperative antibiotic plan in operation notes, the value of interprofessional participation, and the pivotal role of periodic audits to ensure that standards of care have been maintained.
